# Posterior scleritis with choroidal detachments and periaortitis associated with IgG4-related disease: A case report

**DOI:** 10.1097/MD.0000000000029611

**Published:** 2022-07-22

**Authors:** Yoko Mase, Akiko Kubo, Akane Matsumoto, Kosuke Masuda, Masatoshi Kadoya, Kan Koizumi, Chie Sotozono, Mineo Kondo

**Affiliations:** a Department of Ophthalmology, Mie University Graduate School of Medicine, Mie, Japan; b Department of Ophthalmology, Kinan Hospital, Mie, Japan; c Department of Ophthalmology, Japanese Red Cross Society Kyoto Daiichi Hospital, Kyoto, Japan; d Department of Internal Medicine, Kinan Hospital, Mie, Japan; e Department of Rheumatology, Japanese Red Cross Society Kyoto Daiichi Hospital, Kyoto, Japan; f Department of Ophthalmology, Kyoto Prefectural University of Medicine, Kyoto, Japan.

**Keywords:** choroidal detachments, IgG4-related disease, IgG4-related ocular disease, posterior scleritis, periaortitis, Vogt-koyanagki-harada syndrome

## Abstract

IgG4-related diseases (IgG4-RDs) are known to disrupt the functioning of multiple organs and are usually associated with mass lesions. Periaortitis, an inflammation of the adventitia and tissues surrounding the aorta, is an example of an IgG4-RD. In ophthalmology, an enlargement of the lacrimal gland is a well-known IgG4-RD, and scleritis has also been reported to be an IgG4-RD although it is rare. We report our findings in a case with periaortitis and posterior scleritis that were present at the same time, and they responded well to systemic steroid therapy.

**Patients concerns:**

A 79-year-old man with dementia and Lewy bodies was referred to our hospital because of uveitis in both eyes that did not respond to topical steroid therapy.

**Diagnosis:**

We found anterior scleritis in the right eye and uveitis with shallow anterior chambers in both eyes. B-mode echography showed choroidal detachments (CDs) and a T sign in the right eye. The CDs were assumed to have progressed to the posterior scleritis which then caused the severe vision reduction. The patient was referred to the Internal Medicine Department because the systemic inflammatory disease was suspected due to the high levels of C-reactive protein (CRP) and the fast erythrocyte sedimentation rate. Systemic CT scans showed periaortitis only at the lumbar region. Because of the high levels of IgG4, the patient was diagnosed with IgG4-RD.

**Interventions:**

The patient received intravenous and oral steroid therapy. The first 125 mg of methylprednisolone (mPSL) for 3 days was intravenous, after which it was switched to oral prednisolone (PSL) therapy and the dosage was gradually reduced.

**Outcomes:**

The posterior scleritis and periaortitis responded well to the systemic steroid therapy. One year and a half after the onset of the disease, the patient is still taking 5 mg of PSL.

**Conclusions:**

Scleritis with multiple CDs and periaortitis were strongly suspected to be due to IgG4-RD although no definitive diagnosis was made by biopsy of the lesions. Clinicians should be aware that IgG4-RD should be considered as one of the causes of posterior scleritis.

**Key Points**

• Posterior scleritis associated with IgG4-related ocular disease.

• Accumulation of fluid in the posterior episcleral space and extending around the optic nerve, the T sign.

• Shallow anterior chamber with choroidal detachments (CDs).

• Rapid improvements of CDs in response to steroid therapy.

## 1. Introduction

IgG4-related diseases (IgG4-RDs), e.g., autoimmune pancreatitis, can disrupt the functioning of multiple organs and is usually accompanied by mass lesions. Periaortitis, an inflammation of the adventitia and tissues surrounding the aorta, is an example of a condition associated with IgG4-RD. In ophthalmology, a lacrimal gland enlargement is known to be associated with IgG4-RD, and its clinical manifestations include an enlargement of the trigeminal nerve and the external ocular muscles. Posterior scleritis has also been reported to be an IgG4-RD although it is rare.^[1–3]^

Posterior scleritis is an inflammation of the posterior sclera and has a variety of properties including the rapid spread of the inflammation to the adjacent cornea, uvea, optic nerve, external ocular muscles, and eyelids. Thus, posterior scleritis should be diagnosed as quickly as possible to prevent the spread of the inflammation.^[4,5]^

The inflammatory spread of posterior scleritis to the choroid can cause choroidal detachments (CDs), which then hasten the shallowing of the anterior chamber. The signs in these eyes then resemble those of the Vogt-Koyanagi-Harada (VKH) syndrome. Because the treatment protocols of posterior scleritis and VKH syndrome differ, it is important to differentiate them. In addition, the severe CDs with inflammation can make it difficult to differentiate them from choroidal infections and malignant tumors.^[4,5]^ Because of the rapid spread of posterior scleritis, diagnostic steroid treatment is usually performed because a delay can result in poor visual acuity.^[4]^ In spite of the seriousness of IgG4-RD, there is very little information about its characteristics and treatment.

Thus, the purpose of this study was to determine the characteristics of a case with periaortitis and posterior scleritis that were present at the same time. Our results showed that both responded well to systemic steroid therapy.

## 2. Case report

The patient was a 79-year-old man. Two months before his first visit to our clinic, he visited his local ophthalmologist with complaints of decreased vision in both eyes, hyperemia, and pain in the right eye. He was treated with topical and subconjunctival injections of steroids for the uveitis of both eyes. However, there was a rapid deterioration of vision and a shallowing of the anterior chamber in the right eye. He then underwent cataract surgery on the right eye but the shallow anterior chamber did not improve. The patient was then referred to our hospital for further examinations and treatments.

At our initial examination, the right eye had only light perception and the visual acuity of the left eye was 20/63. There was conjunctivitis and anterior scleritis in the right eye and very shallow anterior chambers with posterior synechia in both eyes. Detailed evaluations of the fundus were difficult, and OCT images could not be obtained because of vitreous opacities and miosis. B-mode echography detected choroidal detachments (CDs) and a thickening of the sclera with fluid collecting in the posterior episcleral space that extended around the optic nerve, the T sign, of the right eye.

The patient was referred to the Internal Medicine Department on the same day because systemic inflammatory disease was suspected due to the high levels C-reactive protein (CRP) and fast erythrocyte sedimentation rate (ESR). Systemic CT scan showed only periaortitis at the lumbar region. There were no signs of any other infections.

**Figure 1.**
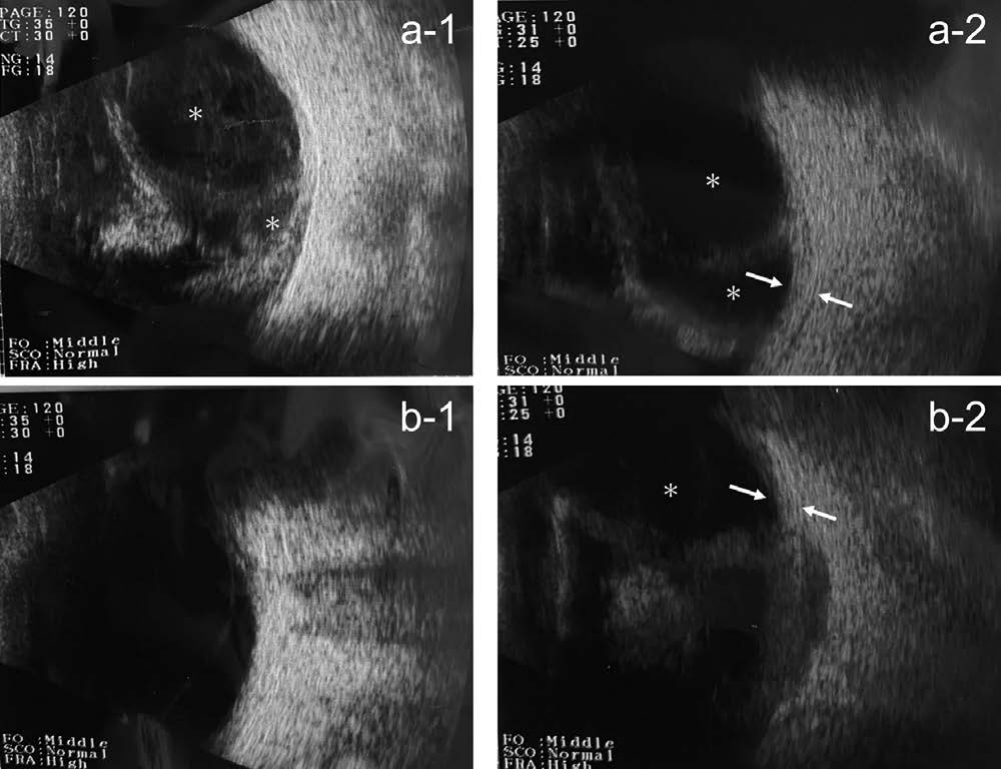
B-scan echographic image of the eyes of a patient with posterior scleritis with choroidal detachments and periaortitis and diagnosed with IgG4-related disease. The 2 top frames are of the right eye. Choroidal detachments (*CDs) are present (left frame (a-1), CDs are growth worth in the right frame (a-2) during 7 days and there is scleral thickening (arrow). Images in the 2 bottom frames are of the left eye, and there is no CD initially (b-1) but after 7 days, CDs (*) appear (b-2) and there is scleral thickening (arrow).

**Figure 2.**
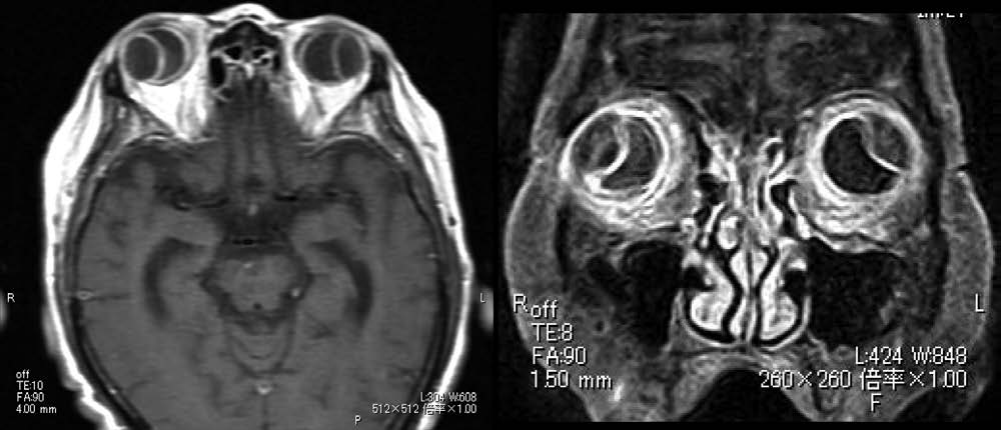
Magnetic resonance images (T1) of both eyes. The CDs and scleral thickening can be seen. There are several CDs and scleral thickening in both eyes.

At the time of the initial examination, there were CDs in only the right eye, but 7 days later, they were also present in the left eye and the visual acuity was also reduced in the left eye (Fig. 1). At this stage, the patient was also found to have high levels of IgG4 (254 mg/dl), and MRI was performed to search for lesions in the orbit. The lacrimal gland was not enlarged, but there were CDs and scleral thickening in both eyes (Fig. 2).

Nineteen days after the initial examination, his right eye did not have light perception and the visual acuity of the left eye was reduced to light perception. Although we suspected a choroidal or orbital tumor initially, we believed that it was more likely to be posterior scleritis because of the subfoveal area of the choroid was less solid and appeared more serous.

We then decided to treat the patient with steroids. Because of the age of the patient, he was treated with intravenous 125 mg of methylprednisolone (mPSL) for 3 days initially and was then switched to 40 mg of oral PSL, and the dose was reduced by 5 mg every week with frequent examinations looking for any signs of a relapse. Then, there was a gradually increase in the examination interval to 2 weeks and then to 1 month, and the treatment has not been completely stopped at this time. In spite of the reduction of the steroid therapy, there were no signs of the scleritis or new CDs.

After beginning the treatment with the corticosteroids, there was a rapid response with the level of c-reactive protein (CRP) decreasing to normal levels after 4 days, and the erythrocyte sedimentation rate (ESR) and IgG4 levels were within normal limits after 1 month. In addition, the inflammation of the aorta was not detected in the CT scan taken after 1 month of steroid therapy.

**Figure 3.**
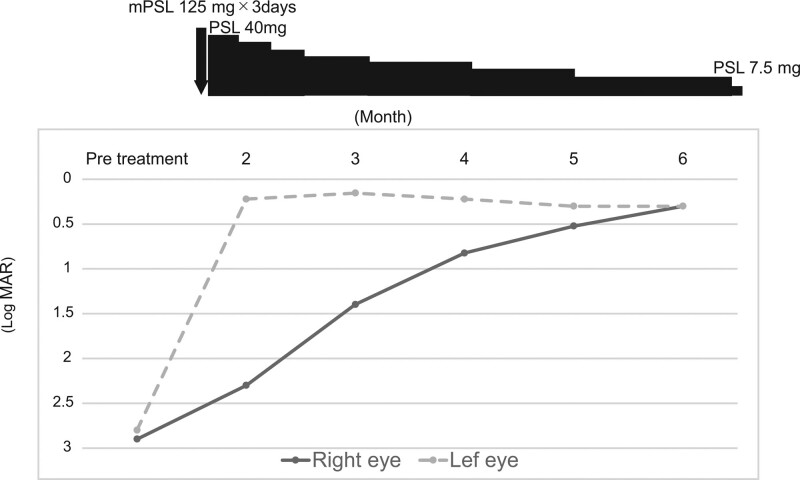
Clinical course of visual acuity and steroid therapy. The visual acuity of the left eye has improved and that of the right eye slowly improved in response to the steroid treatment.

During the course of the disease process, various examinations were performed to confirm the diagnosis and that no other abnormalities were present. B-mode echography showed improvements of the CDs, and the visual acuity gradually improved to normal levels (Fig. 3).

**Figure 4.**
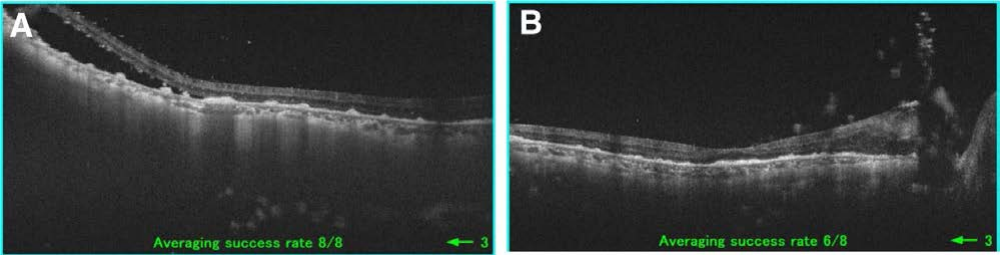
Optical coherence tomographic images. A: 5 months after beginning the treatment, there are subretinal deposits and serous retinal detachment in the right eye. B: Reducing the dose of prednisolone (PSL) led to a reduction and gradual improvement of the subretinal deposits. No choroidal thickening can be seen.

Five months after the onset of the signs and symptoms of posterior scleritis, OCT imaging became possible because of the disappearance of the vitreous opacities. The OCT images showed subretinal deposits and serous retinal detachment in the right eye (Fig. 4A). No choroidal thickening was observed. Thereafter, the serous detachment gradual improved and the subretinal deposits were reduced (Fig. 4B).

## 3. Discussion and Conclusions

Patients with IgG4-RD present with tissue infiltration and formation of IgG4-positive plasma cells that form masses in various organs of the body. This disease was initially diagnosed as autoimmune pancreatitis in 2001.^[6]^ In ophthalmology, Mikulicz disease was first reported to be an IgG4-RD in 2004.^[7]^ In 2011, the Ministry of Health, Labor and Welfare of Japan developed the comprehensive diagnostic criteria of IgG4-RDs.^[8]^

The pancreas is the organ most common affected by IgG4-RD followed by the salivary glands, kidneys, lacrimal glands, periaortitis, bile ducts, and lungs.^[9]^ An enlargement of the lacrimal gland is a well-known IgG4-related ocular disease (IgG4-ROD), but posterior scleritis, uveitis, and optic neuropathy have also been reported although they are relatively rare.^[2,3]^ Scleritis and uveitis due to IgG4-ROD was first reported in 2012.^[3]^

Philippakis et al reported the first case of conjunctival carcinoma in a patient with IgG4-RD scleritis,^[10]^ but there have been only a few reports of IgG4-related scleritis in the PubMed data base.

Although lacrimal gland enlargement is a well-known IgG4-ROD, it was reported that 52.3% of patients with IgG4-ROD have lesions in organs other than the lacrimal glands.^[11]^ In our case, no definitive diagnosis was made by biopsy of the sclera and the periaortic tissue because of its difficulty but IgG4-RD was strongly suspected based on the imaging and serological findings.^[8]^

**Figure 5.**
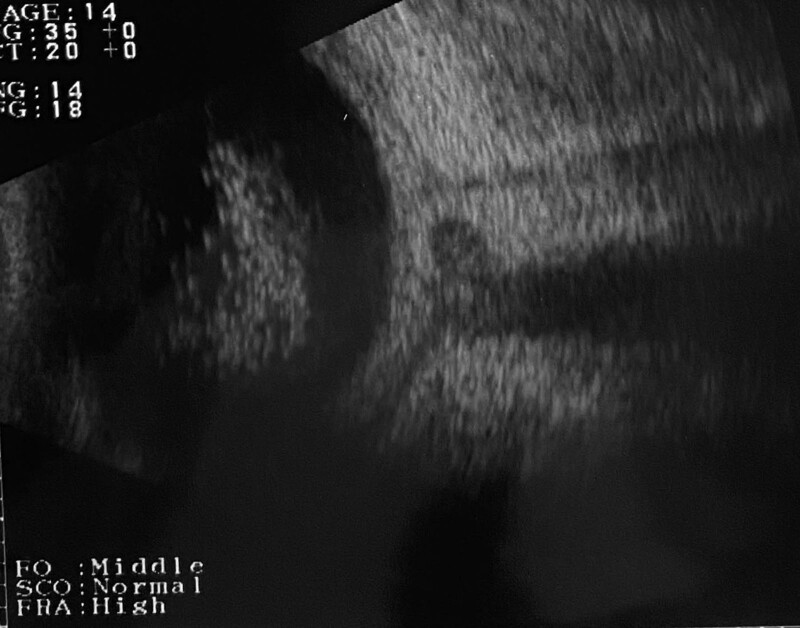
Presence of T-sign. The fluid distends the tenon capsule and space with peri-optic edema because of severe inflammation of the sclera.

Autoimmune diseases have been reported to be the cause of many diseases,^[12,13]^ and IgG4-RD was suspected in our case. When scleritis is accompanied by multiple CDs and shallow anterior chamber, it is difficult to differentiate it from VKH disease. B-scan ultrasonography was important in the differential diagnosis of posterior scleritis because the images showed the pathognomonic T-sign due to fluid collecting in the posterior episcleral space and extending around the optic nerve (Fig. 5).^[14]^ In our case, the T-sign was present from the time of the initial examination especially in the right eye, and even after the improvement of the anterior scleritis, vitreous opacity, and CDs, the T-sign was still present. We assumed that the right eye was more affected by the severe scleritis than the left eye, and this may be the reason why the visual acuity of his right eye improved more slowly.

The scleritis with the CDs was strongly suspected to be an IgG4-ROD because of the presence of high levels IgG4, scleritis, and periaortits at same time, although no definitive diagnosis was made by biopsy of leasions. IgG4-RD should be considered as one of the possible causes of the posterior scleritis although it is relatively rare. And it is also important to consider that posterior scleritis may also be a subretinal or choroidal mass, and it may be difficult to differentiate inflammatory lesions from an intraocular tumor based on imaging alone.^[4]^ Early systemic search and cooperation with the Internal Medicine Department is important because IgG4-RD is a chronic inflammatory condition that may involve nearly every organ system. It should be noted that not only a monitoring of local recurrences but also screening intertemporal development in other organs would be important.

## Acknowledgment

We thank Professor Emeritus Duco Hamasaki of the Bascom Palmer Eye Institute of the University of Miami for critical discussion and final manuscript revisions.

## Author contributions

Yoko Mase: Conceptualization, Writing-Original draft preparation; Akiko Kubo: Conceptualization, Writing-Reviewing and Editing; Akane Matsumoto: Writing-Reviewing and Editing; Kosuke Masuda: Visualization, Writing-Reviewing and Editing; Masatoshi Kadoya: Writing-Reviewing and Editing; Kan Koizumi: Writing-Reviewing and Editing; Chie Sotozono: Writing-Reviewing and Editing; Mineo Kondo: Project administration, Writing-Reviewing and Editing.
